# Spatial distribution patterns of plague hosts: point pattern analysis of the burrows of great gerbils in Kazakhstan

**DOI:** 10.1111/jbi.12534

**Published:** 2015-05-19

**Authors:** Liesbeth I. Wilschut, Anne Laudisoit, Nelika K. Hughes, Elisabeth A. Addink, Steven M. de Jong, Hans A.P. Heesterbeek, Jonas Reijniers, Sally Eagle, Vladimir M. Dubyanskiy, Mike Begon

**Affiliations:** ^1^ Department of Physical GeographyUtrecht UniversityUtrechtThe Netherlands; ^2^ Faculty of Veterinary MedicineUtrecht UniversityUtrechtThe Netherlands; ^3^ Ecology Evolution and Genomics of Infectious Disease Research GroupInstitute of Integrative BiologyThe University of LiverpoolLiverpoolUK; ^4^ Evolutionary Ecology GroupDepartment of BiologyUniversity of AntwerpAntwerpBelgium; ^5^ Stavropol Plague Control Research InstituteStavropolRussian Federation

**Keywords:** Bubonic plague, clustering, desert, GIS, infectious disease, landscape epidemiology, *Rhombomys opimus*, rodents, spatial point pattern analysis, *Yersinia pestis*

## Abstract

**Aim:**

The spatial structure of a population can strongly influence the dynamics of infectious diseases, yet rarely is the underlying structure quantified. A case in point is plague, an infectious zoonotic disease caused by the bacterium *Yersinia pestis*. Plague dynamics within the Central Asian desert plague focus have been extensively modelled in recent years, but always with strong uniformity assumptions about the distribution of its primary reservoir host, the great gerbil (*Rhombomys opimus*). Yet, while clustering of this species’ burrows due to social or ecological processes could have potentially significant effects on model outcomes, there is currently nothing known about the spatial distribution of inhabited burrows. Here, we address this knowledge gap by describing key aspects of the spatial patterns of great gerbil burrows in Kazakhstan.

**Location:**

Kazakhstan.

**Methods:**

Burrows were classified as either occupied or empty in 98 squares of four different sizes: 200 m (side length), 250 m, 500 m and 590–1020 m. We used Ripley's *K* statistic to determine whether and at what scale there was clustering of occupied burrows, and semi‐variograms to quantify spatial patterns in occupied burrows at scales of 250 m to 9 km.

**Results:**

Significant spatial clustering of occupied burrows occurred in 25% and 75% of squares of 500 m and 590–1020 m, respectively, but not in smaller squares. In clustered squares, the clustering criterion peaked around 250 m. Semi‐variograms showed that burrow density was auto‐correlated up to a distance of 7 km and occupied density up to 2.5 km.

**Main conclusions:**

These results demonstrate that there is statistically significant spatial clustering of occupied burrows and that the uniformity assumptions of previous plague models should be reconsidered to assess its significance for plague transmission. This field evidence will allow for more realistic approaches to disease ecology models for both this system and for other structured host populations.

## Introduction

The social or spatial structure of wildlife populations has significant implications for the invasion (Keeling, 1999), spread (Grenfell *et al*., 2001) and persistence of infectious diseases (Keeling & Gilligan, 2000). Far from being uniformly distributed in the environment, many wildlife populations resemble a metapopulation, i.e. a network of subpopulations that are connected to varying degrees by temporary movements or dispersal of individuals (Hanski, 1999; Leibold *et al*., 2004). This network structure has significant implications for the spread of an infection, as transmission within a population depends on group size, the frequency of individual movements between groups, and the time the disease persists within each group (Grenfell & Harwood, 1997; Keeling & Gilligan, 2000; Keeling & Rohani, 2002; Cross *et al*., 2007; Jesse & Heesterbeek, 2011). The size and structure of the subpopulation network, and the extent to which subunits are clustered (relative to host movements) also influence the dynamics and persistence of an infection (Bolker, 1999): infections are less likely to persist in small populations that are not well connected, than they are in large or well‐connected populations. Knowledge of the underlying spatial structure of wildlife populations is therefore key to understanding and modelling how an infectious disease spreads through host populations.

One disease for which the spatial structure of host populations is likely to be very important is plague. Caused by the bacterium *Yersinia pestis*, plague is naturally resident in rodent populations throughout much of the world (Gage & Kosoy, 2005), and still causes 1000–3000 human cases each year, predominantly in Africa (World Health Organization, 2004). The disease has received a resurgence of scientific interest in recent years, as it is the first disease for which wildlife abundance thresholds have been confirmed (Davis *et al*., 2004; Reijniers *et al*., 2012). Much of this work has focused on the Central Asian desert plague focus in south‐east Kazakhstan, where the burrowing great gerbil, *Rhombomys opimus* (Lichtenstein, 1823), is the main host and *Xenopsylla* spp. fleas are the primary vectors. Abundance thresholds exist here for both the pathogen's host (Davis *et al*., 2004) and vectors (Reijniers *et al*., 2012). The host abundance threshold has been shown to be a percolation threshold, suggesting that a level of connectedness between occupied burrows is required for plague to spread (percolate) across the landscape (Davis *et al*., 2008). Importantly, models of this plague system have excluded information on the population structure, and have instead assumed a homogeneous or random distribution of hosts (Davis *et al*., 2004; Schmid *et al*., 2012). However, the validity of these assumptions has not been established, although deviations from these assumptions are likely to modify model outcomes by hastening or slowing the spread of plague across the landscape.

Here, we analyse field data on the burrow locations of great gerbils to determine the spatial distribution of the gerbil metapopulation, and thus the likely implications for the spread of plague throughout gerbil populations. Several aspects of the ecology of great gerbils make them highly suited for such analyses. Great gerbils are social rodents that live in family groups in large underground burrows (Randall *et al*., 2005), which can remain in the landscape for many years. Because of large fluctuations in the size of the gerbil population (Davis *et al*., 2004), not all burrows are consistently occupied; some burrows may remain empty for several years at low population density until population density rises again and a burrow is re‐occupied by a migrating gerbil. The transmission of plague within a burrow occurs relatively easily: infected fleas actively move within the burrow and hence great gerbils within a burrow tend to have the same disease status (Davis *et al*., 2007). Fleas cannot survive outside a burrow except on a host, however, and flea colonization of new burrows and hosts depends on direct contact between individual great gerbils from different burrows, or on visits to other burrows. Critical to the spread of fleas and plague, therefore, are the movements of great gerbils, and, to a lesser extent, of other hosts, between burrows. Daily movements are, however, short in duration and distance, and 90% of the time is spent at their home burrow (Rogovin *et al*., 2003). There are thus possibly strong spatial constraints to the transmission of plague that depend on population size and the spatial distribution of occupied burrows.

**Figure 1:**
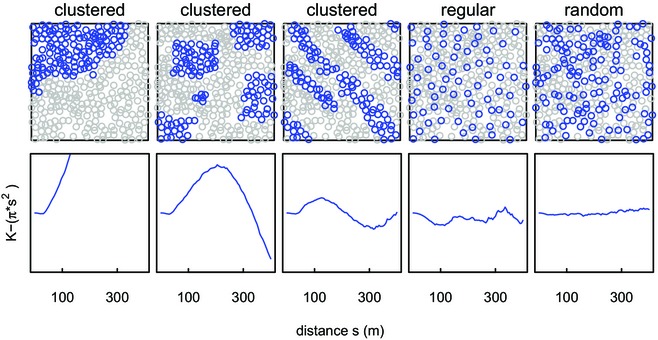
Simulated distribution patterns of great gerbil (*Rhombomys opimus*) burrows and their corresponding Ripley's *K*. Top row: examples of possible distributions of occupied burrows (blue/dark circles) against a background of unoccupied burrows (grey circles; 335 burrows in total), including clustered (first three figures), regular and random distributions. Bottom row: corresponding Ripley's *K* graphs for each distribution (plotted as *K* − π*s* ^2^ for clarity). Ripley's *K* (Ripley, 1976) is a commonly used statistical method to gain insight into point patterns, where clustered patterns give larger values of Ripley's *K* than regular or random patterns.

We consider three possible distributions of occupied burrows that may affect the transmission dynamics of plague: random, clustered or regular (Fig. 1). The significance of the distributions for plague models is that plague would be able to spread more easily within clusters, but that the chance of plague transmitting among clusters is lower, although this depends on the configuration of the clusters relative to gerbil movements.

Spatial clustering of great gerbil families (occupied burrows) could occur by several mechanisms. First, exogenous factors such as climate and topography may result in higher or lower quality habitat patches for gerbils (Naumov & Lobachev, 1975). For example, if higher soil moisture improves gerbil survival, great gerbils are likely to persist and cluster at those locations where soil moisture is higher. Second, spatial clustering of occupied burrows may also arise due to interactions between the great gerbils themselves, such as reproductive needs or localized juvenile dispersal (Hanski, 1999).

The objective of this study was to analyse, by spatial point pattern analysis, both the spatial distribution of all burrows, irrespective of occupancy, and of occupied and empty burrows. To do so, we collected data on the spatial distribution and occupancy status of burrow systems in a variety of landscapes, and in 98 research squares of varying sizes. Also, in order to study the spatial structure at scales larger than 250 m, semi‐variograms were used to examine the spatial autocorrelation of burrow and occupied burrow densities.

Specifically, we address the following questions. Do the burrows, occupied burrows and empty burrows have an aggregated pattern? If the occupied and/or empty burrows are spatially clustered, at what spatial scale can this be detected? Is spatial clustering of occupied burrows related to landscape‐ecological variables? And finally, to what extent is there auto‐correlation in the burrow densities and the occupied burrow densities at scales of 250 m to 9 km?

## Materials and Methods

### Study area

**Figure 2:**
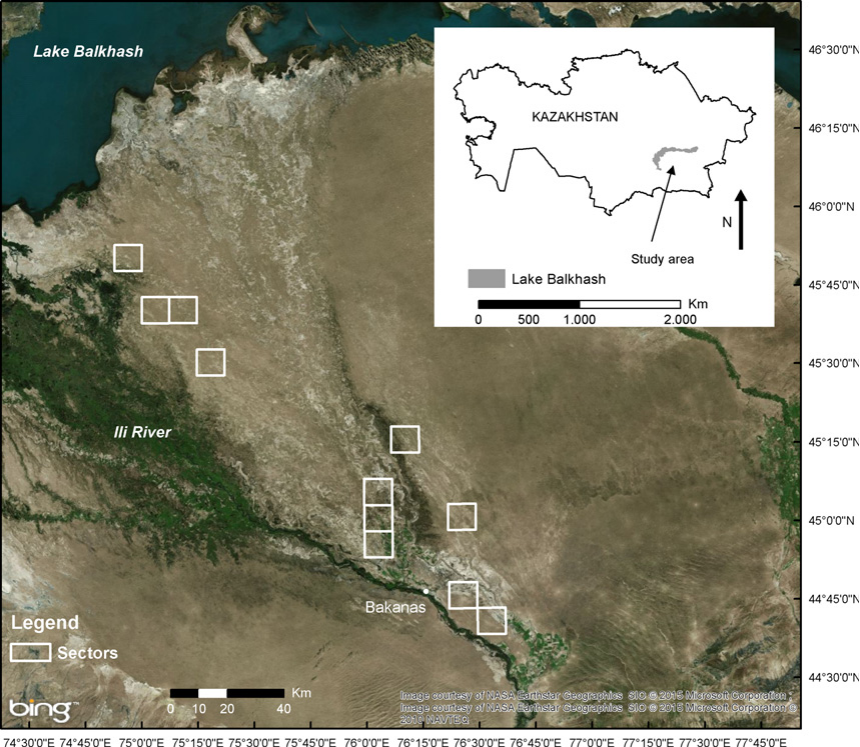
The study area in Kazakhstan in which the distribution of great gerbil (*Rhombomys opimus*) burrows was quantified. Burrows were mapped in 98 research squares within 11 sectors (white squares). The inset shows the location of the study area in Kazakhstan. Source: Bing Maps, 2011.

The study area is a semi‐arid area located in south‐eastern Kazakhstan, south of Lake Balkhash (Fig. 2). The Ili River provides the main source of moisture for the area. Burrows of the great gerbil within the study area are abundant, with an average burrow density of *c*. 3.5 burrows ha^−1^ (Wilschut *et al*., 2013a). The mean diameter of burrows varies from *c*. 20 m in the floodplain to 35 m in the dunes.

Three main landforms compose the landscape in the study area: the active river, floodplains and dunes. Soils are sandy, with variable clay and low organic matter content. Overall, vegetation cover is low and has a decreasing trend towards Lake Balkhash (Wilschut *et al*., 2013a).

### Field data

Data collection was carried out inside research squares of varying side lengths such that the effect of the size of the research area on the spatial point pattern analysis could be determined. The squares were monitored in different seasons and years to sample across a range of occupancies. All burrows inside the squares were mapped, deriving coordinates from a GPS at the ‘ecological centre’ of the burrow – the place of most intensive great gerbil activity. The occupancy status of each burrow, either occupied or empty, was determined from signs of foraging activity, presence of fresh faeces and/or scent (urine) marks and the recent clearance of burrow entrances (indicated by the presence of freshly dug sand).

All field data were collected inside 11 ‘sectors’ (Fig. 2), which are monitoring units of *c*. 10 km × 10 km (Wilschut *et al*., 2013b). The data were collected within 98 squares (Table 1). The placement of these squares was done by randomly selecting several locations per sector (as far as accessibility allowed, because roads are scarce), so that variability within the sectors was covered. At each location, one to five squares were laid out, with a minimum distance of 200 m between squares. Data were collected at four spatial scales.

**Table 1:** Overview of all squares monitored in the research area in Kazakhstan. In every square, burrows of the great gerbil (*Rhombomys opimus*) were marked with a GPS and their occupancy status (occupied or empty) was recorded

Side length squares	No. of squares [no. of squares visited more often]	No. of times visited	Total no. of square visits	No. of burrows in the squares	Total no. of burrows in all squares of all visits	No. of squares with > 10 burrows	No. of squares with > 10 occupied burrows	No. of squares with > 10 empty burrows
200 m	36 [6]	6 +2	216 +12	475	2996	204	27	98
250 m	38	2	76	672	1344	69	15	55
500 m	16 [9]	1 +1	16 +9	1403	2257	25	24	25
590–1020 m	8	1	8	1457	1457	8	8	8
Total	98	–	337	4007	8054	306	74	186

First, 36 squares of 200 m (side length) were monitored during six field sessions: April, June and September in 2011 and 2012. In one of the sectors the squares were also monitored in April 2013 and September 2013 (i.e. eight seasons in total). These 36 squares are spread evenly over six sectors (i.e. six per sector).

Second, 38 squares of 250 m (side length) were mapped in April 2013 and September 2013 in one of the sectors. Placement of the squares was done in a similar way as the 200 m squares. Sixteen squares measuring 500 m were also visited: five in September 2011, five in June 2012, five in September 2012 and another one in May 2013. Nine of those squares were visited twice. Finally, eight squares, of which two had a rectangular shape, of varying sizes between 590 and 1020 m were monitored in May 2013.

### Spatially clustered and aggregated distributions

In this study two types of point patterns are analysed: marked point patterns and unmarked point patterns (Diggle, 2003; Lancaster & Downes, 2004; Atkinson *et al*., 2007). In an unmarked point pattern there is only one type of point, and using point pattern analysis one can study to what extent these points (e.g. burrows) are aggregated. A marked point pattern is a subset of points with a common attribute within a complete background set of points (e.g. occupied burrows among all burrows). This allows for testing of clustering of marked points, given the distribution of all points.

Our first aim was to gain some general insight into the burrow distribution, regardless of occupancy. To do so, we calculated whether or not burrows were aggregated by comparing their distribution with completely spatial random patterns. This was repeated for occupied and empty burrows. The second aim was to determine whether the occupied burrows were significantly clustered. To do this, we calculated spatial clustering for both occupied and empty burrows, among the overall distribution of burrows. The third aim was to evaluate whether there was a relationship between spatial clustering of occupied burrows and landscape‐ecological variables.

#### Testing for spatial aggregation and spatial clustering of burrows using Ripley's *K*

We used Ripley's *K* (Ripley, 1976), hereafter referred to as *K*, for both spatial aggregation and spatial clustering analyses. *K* is given by:

$$K(s)=\lambda^{-1}n^{-1}\sum_{i=1}^{n}\sum_{j\neq 1}I\left(d_{ij}<s\right)$$

where *s* is the distance, *I*, the indicator function is equal to 1 if *d*_*ij*_ is less than or equal to *s*, otherwise it is zero. *n* is the total number of points, λ is the average density of points (*n/A* where *A* is the surface area of the region containing all points), and *d*_*ij*_ is the Euclidean distance between point *i* and point *j*.

For clarity, *K* − π*s* ^2^ was plotted instead of *K* (Diggle, 2003), as *K* − π*s* ^2^ is scale independent.

The advantage of *K* over other methods, for example nearest neighbourhood methods, is that it is independent of the density of the point pattern (Gatrell *et al*., 1996) and thus can describe the spatial point patterns at many different scales (Dixon, 2002).

#### Testing for spatial aggregation of burrows using complete spatial randomness (csr)

First, we used the *K* statistic to investigate to the extent to which all burrows, the occupied burrows and the empty burrows, have an aggregated distribution. For all squares, we calculated the *K*(*s*) statistic for all burrows (*K*_all_) using the R package splancs (Rowlingson & Diggle, 2013). *K*(*s*) was calculated discretely: every 5 m up to 175 m for the 200 m and 250 m squares and every 5 m up to 400 m for the larger squares. To assess which *K*‐values can be expected from a random distribution, we generated a complete spatial random distribution of burrows (csr) with the same burrow density (using the r package spatstat; Baddeley & Turner, 2005) and calculated its corresponding *K*_crs_ curve. We repeated this 1000 times and calculated the upper (*K*_csr_97.5_) and lower (*K*_csr_2.5_) confidence interval curves from the ensemble of curves. If for a particular distance *s K*_all_(*s*) is larger than *K*_csr_97.5_(*s*) (or conversely, lower than *K*_csr_2.5_(*s*)), this suggests that burrows within a distance *s* are aggregated (or conversely, regularly spaced). Next, for all distances, the results for all squares were summarized by calculating the percentages where *K*_all_ > *K*_csr_97.5_ or *K*_all_ < *K*_csr_2.5_. The procedure was repeated for the occupied and the empty burrows, denoting the *K*(*s*) statistics as *K*_occ_ and *K*_emp_, respectively.

To investigate the relationship between the size of the research area and spatial aggregation, first the frequencies of occurrence of aggregation for each size group of squares (200 m, 250 m, 500 m and 590–1020 m) were calculated. The *K*_all_, *K*_occ_ and *K*_emp_ patterns within an individual square were defined as being aggregated when the specific *K* was larger than *K*_crs_97.5_ for a distance of at least 35 m; this value was based on the mean nearest burrow neighbour distance (38.49 m) calculated using the 33 largest squares.

#### Testing for spatial clustering of occupied and empty burrows using random sampling (rs)

Spatial clustering is defined as a general tendency for a marked point pattern (here, that of *occupied* or *empty* burrows) to occur more closely together than would be expected from rs from within the ‘total population’ (here, *all* burrows) (Diggle, 2003). To examine this, *K*(*s*) was calculated using only the occupied burrows (*K*_occ_), and the confidence interval was simulated in a similar way as stated earlier, but now by randomly selecting (rs) the observed number of occupied burrows from the locations of all burrows. Because of the measure's sensitivity to small sample sizes (Wiegand & Moloney, 2004), *K* was calculated only when the number of occupied burrows was greater than 10 (see Table 1). As stated earlier, the percentages of squares were calculated where *K*_occ_ > *K*_rs_97.5_ or *K*_occ_ < *K*_rs_2.5_. The frequencies of occurrence of spatial clustering for each size group were calculated as well. The procedure was repeated for the empty burrows.

Generalized linear models (GLMs) were then used to test whether there was a significant relationship between the presence/absence of spatial clustering of occupied burrows and square size, number of burrows, number of occupied burrows and occupancy level. Models were bootstrapped to account for the repeatedly visited sites: each model was run 100 times, each time using all research locations, but randomly drawing one value from the sites that were visited multiple times. The minimum and maximum *P*‐values were then extracted to see whether there was a significant influence of the repeatedly visited sites on the model outcome.

#### Evaluating the relation of spatial clustering and landscape‐ecological variables

GLMs were also used to test whether there was a relationship between clustering and landscape‐ecological variables, as well as variables related to the research design, such as the season of data collection (i.e. spring, summer or autumn). The presence or absence of clustering was used as a dependent variable, while explanatory variables included landscape type, presence of dunes (both derived from a landscape map; Wilschut *et al*., 2013a), latitude and longitude, season of data collection, number of occupied burrows and occupancy level. Landscape types were steppe on inactive floodplain, steppe on inactive floodplain with salt flats, active floodplain with shrub‐dominated vegetation and floodplain with developing vegetation, and there were four types of dunes: dynamic dunes, stabilized dunes with inactive vegetation, stabilized dunes with active vegetation on moist soil and stabilized dunes with active vegetation (Wilschut *et al*., 2013a). The Akaike information criterion (AIC) (Akaike, 1974) was used to determine the relative support for each model. The best model was selected by first selecting the models with the lowest AIC values. Between models with comparable explanatory power (ΔAIC < 2), the simplest was chosen (applying the principle of parsimony). Only the squares from the size groups where detection of spatial clustering proved to be possible were used.

### Calculating the spatial auto‐correlation of burrow density and occupied burrow density

Although *K* provides information on the distribution of (occupied and empty) burrow point patterns up to a distance of 400 m, larger scale spatial patterns can also be important to incorporate into plague persistence models. Therefore, we used the density of burrows and the density of occupied burrows to construct semi‐variograms (Burrough & McDonnell, 1998; Oliver & Webster, 2014), in order to determine whether there is spatial autocorrelation of (occupied) burrow densities at a larger scale, and also to detect at what distance the autocorrelation ceases. Semi‐variograms were calculated for the data collected in the spring of 2013 only, as this was the season in which the greatest number of squares was monitored. Squares of 500 m and larger were first subdivided into multiple 250 m squares, and the burrow density per square, as well as the occupied burrow density per square, was then calculated for all squares (133 in total). The centres of the squares were used as the point location. The maximum distance between the centres of two squares was 180 km and the minimum distance was 250 m. However, a cut‐off value of 9 km was used because large gaps existed between the sectors within which data were collected. The occupied density values were first log‐transformed because the frequencies had a slightly skewed distribution. Semi‐variograms were then constructed and fitted using the R package gstat (Pebesma, 2004).

## Results

### Aggregated and regular patterns of burrows – comparison with csr

**Figure 3:**
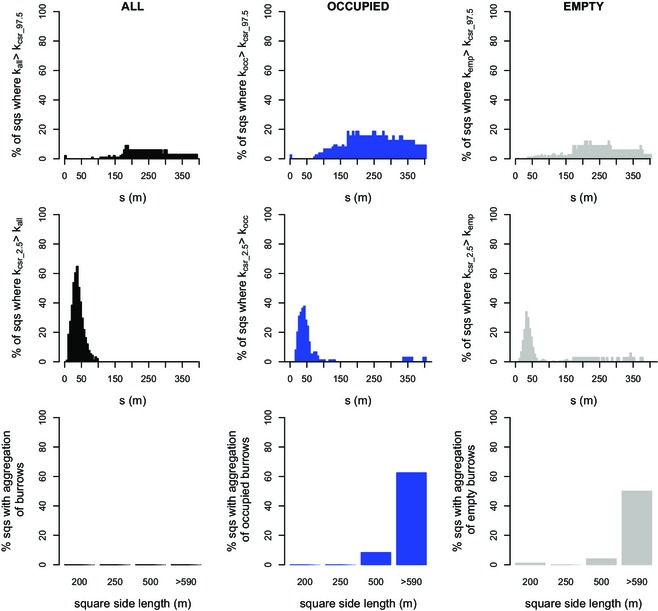
The spatial distribution of great gerbil (*Rhombomys opimus*) burrows as a function of distance. Top row: the percentage of squares (sqs) where the burrows had a significantly aggregated pattern (*K*_all_ > *K*_csr_97.5_) as a function of distance *s*. Middle row: the percentage of squares where the burrows had a significantly regular pattern (*K*_csr_2.5_ > *K*_all_) as a function of distance *s*. Bottom row: the percentage of squares that were classified as aggregated, grouped per size group. Data are shown for all burrows (left column), occupied burrows (middle column) and empty burrows (right column).

In only a few cases was aggregation identified for *all* burrows (maximally 9% of squares at *s* = 185 m), and then only for *s* > 105 m (Fig. 3, top left). Aggregation was more common for occupied and empty burrows, however, and especially at distances greater than *s* = 175 m (occupied maximally 22% at *s* = 200 m and empty maximally 12% at several distances between 210 and 285 m; Fig. 3).

Regular patterns were found at small distances up to about 40 m, where most burrow patterns were evenly distributed (*K*_csr_2.5_ > *K*_all_) compared to completely random point patterns (Fig. 3, middle row). Occupied and empty burrows showed a similar, although less pronounced, pattern.

Characterizing *K*_all_ patterns in individual squares showed that none of them were significantly aggregated (Fig. 3, bottom row). The occupied burrow patterns (*K*_occ_), however, did show aggregation: in 62.5% of the squares in the largest size group and in 8.3% of the squares in the 500 m size group, the patterns were classified as significantly aggregated (i.e. for a distance of at least 35 m *K*_occ_ is larger than *K*_csr_97.5_). For the empty patterns (*K*_emp_), the percentages of squares that were aggregated were 50% and 4%, respectively.

### Spatial clustering of occupied and empty burrows – comparison with rs

**Figure 4:**
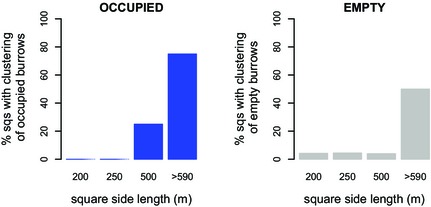
The clustering of occupied and empty burrows of the great gerbil (*Rhombomys opimus*) in research squares (sqs) of different side lengths in Kazakhstan. For each size group, the percentage of squares in which occupied (left) and empty (right) burrows were clustered is given. The distribution of occupied and empty burrow was analysed given the distribution of all burrows.

Of a total of 74 tested squares, significant spatial clustering of occupied burrows (Fig. 4, left) occurred in 12 squares (16.2%), 6 of which were in the largest size group (75% of squares > 590 m) and 6 were in the 500 m squares (25%). No significant clustering was detected in any of the 250 m or 200 m squares. Examples of the distribution of occupied burrows in both a clustered and not clustered square are shown in Appendix S1: Fig. S1 in Supporting Information.

Overall, empty burrows were clustered less often than occupied burrows (5.9% of squares; Fig. 4, right), but clustering again varied with square size: 50% of the largest squares showed clustering, while only 4% of the 500 m squares did so.

GLMs revealed that square size was significantly positively correlated with clustering (*P* = 0.002, Appendix S1: Table S1) and that research areas should be at least 580 m (size length) to detect spatial clustering with a chance of more than 50% (Appendix S1: Fig. S2). The model was not improved by including either the number of burrows or the number of occupied burrows (Table S1), while bootstrapping the model resulted only in a very slight difference in the maximum and minimum *P*‐values. Hence, the model was run with all squares available.

**Figure 5:**
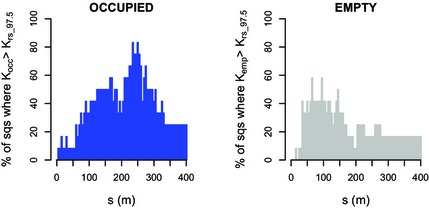
Analysis of burrows patterns of the great gerbil (*Rhombomys opimus*) in Kazakhstan, in research squares (sqs) that were classified as clustered. The percentage of clustered squares where occupied (left) and empty (right) burrows were classified as clustered (*K*_occ_ > *K*_rs_97.5_) at a certain distance (*s*).

Focusing on the squares classified as clustered (Fig. 5), for distances of 130 m and above, the percentage of squares that had a *K*_occ_ larger than *K*_rs_97.5_ was often 50% or more, peaking at around 250 m in *c*. 80% of the squares. The distance where clustering of empty burrows was at its peak (at *s* = 70–100 m in *c*. 50% of squares) was smaller.

### Spatial clustering and the relationship with landscape‐ecological variables

To test whether absence or presence of spatial clustering could be explained by the landscape, several GLMs were constructed for the squares of the two largest size groups, because in those squares clustering proved to be possible to detect. Similar to the GLMs including all squares, the most parsimonious model here also included only square size, even though the 33 squares in the model were already selected on the basis of size (*P* = 0.065, Table 2). None of the landscape‐ecological or other variables contributed significantly to the model.

**Table 2:** In Kazakhstan, clustering of occupied burrows of the great gerbil (*Rhombomys opimus*) was detected in 12 squares of the two largest size groups. Several models were tested to see whether landscape‐ecological variables were correlated with the absence and presence of spatial clustering of occupied burrows. Results of these GLMs for the research squares from the two largest size groups show that landscape‐ecological variables do not contribute significantly to the models. The five models with the lowest Akaike information criterion (AIC) values are shown, ranked by AIC

Variable	Intercept	Coefficient	*P*	Variable 2	Coefficient	*P*	AIC
Size (m^2^)	−3.24	8.7.2e‐06	0.065	–	–	–	38.4
No. of burrows	−2.72	0.02	0.1				39.9
Size (m^2^)	−3.01	1.66e‐05	0.07	No. of occupied burrows	−1.6e‐02	0.52	39.9
Size (m^2^)	−2.7	8.6e‐06	0.08	% occupancy	−1.0e‐02	0.7	40.2
Size (m^2^)	−3.3	8.7e‐06	0.07	Presence of dunes	9.9e‐02	0.9	40.4

### Spatial auto‐correlation of burrow density and occupied burrow density

**Figure 6:**
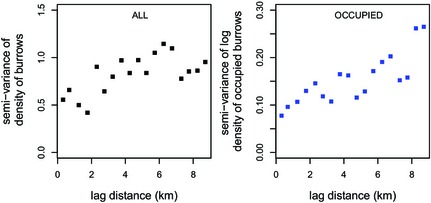
Semi‐variograms of great gerbil (*Rhombomys opimus*) burrow densities in the research area in Kazakhstan. Left: Semi‐variogram of the density of all burrows (both occupied and empty burrows). Right: Semi‐variogram of the log density of occupied burrows.

In total, 133 data points were available in April 2013, from which a burrow density and an occupied burrow density semi‐variogram were constructed. The density values in the squares varied between 0.6 and 5.4 burrows ha^−1^ (mean: 3.2 burrows ha^−1^); histograms are given in Appendix S1: Fig. S3. While burrows (irrespective of occupancy) showed no aggregation within the squares, differences in burrow density were apparent between squares. The semi‐variogram of burrow densities (Fig. 6, left) showed burrow densities were auto‐correlated up to *c*. 7 km (spherical fit, range = 7.2 km).

The density of occupied burrows varied between 0 and 3.0 per hectare. The maximum distance at which the occupied burrow densities were auto‐correlated was 2.5 km (spherical fit, range = 2.5 km).

## Discussion

For plague in great gerbils, it has been established that a gerbil‐density threshold (percentage of burrows occupied) is required for plague to spread and persist (Davis *et al*., 2004). That this is a ‘percolation’ threshold shows that there is a level of connectedness between occupied burrows required for plague to spread across the landscape (Davis *et al*., 2008). The level of connectedness will depend strongly on the configuration of the occupied burrows. To date, models have assumed a random or regular distribution of occupied burrows, such that an increase in occupancy will increase the connectedness gradually (assuming, too, a homogeneous population density across the plague focus). Here, we have established instead that occupied burrows are clustered locally. These results suggest that both the invasion and the persistence of plague will be influenced not only by the distances between occupied burrows but also by the distances between clusters of occupied burrows.

More specifically, it is broadly understood that the dynamics of an infectious disease may be affected if a population exhibits a ‘metapopulation’ structure: a series of subpopulations linked by migration, but within each of which disease invasion and extinction are more important than detailed rises and falls in prevalence (e.g. Grenfell & Harwood, 1997; Jesse & Heesterbeek, 2011). For example, migration in metapopulations can either increase or decrease the chances of overall disease‐extinction, depending on details of the species biology (Harding *et al*., 2012). However, the scale at which subpopulations are defined is open to question. Thus, for plague in great gerbils in Kazakhstan, Davis *et al*. (2007) argued in favour of the individual burrow system being seen as a subpopulation. The current results, however, suggest that clusters of occupied burrows may be the more appropriate unit, with knock‐on consequences for the scale at which migration and connectedness should be measured. As a more general point, these results remind us that ‘patchiness’ in spatial host–pathogen systems is the norm rather than the exception. Spatial host–pathogen systems should be assessed to consider whether the appropriate scale of the subpopulation is the most obvious physical structure (e.g. the burrow, the habitat patch, the individual plant) or whether patterns of host occupation or abundance make it more appropriate for these to be grouped into clusters.

To understand the spatial distribution of occupied burrows, it is helpful to first study the distribution of *all* burrows, i.e. irrespective of occupancy. As burrows typically remain for many years in the landscape, the current overall burrow pattern is a reflection of where gerbils are capable of constructing burrows, and perhaps particularly of peak gerbil densities when all burrows are occupied, presuming that all suitable habitats contain burrows. We found that at small distances (< 40 m), burrows often show significant spatial regularity, confirming earlier findings using nearest neighbourhood statistics (Wilschut *et al*., 2013a). This regular pattern can be explained by the territorial nature of great gerbils, and the diameter of the burrow itself, which is on average between 20 and 35 m (Naumov & Lobachev, 1975; Rogovin *et al*., 2003; Wilschut *et al*., 2013a). Little evidence of aggregation of burrows was found at distances up to 400 m.

There was evidence, however, of aggregation (compared to a random distribution) of occupied (and empty) burrows. This is also reflected in the patterns of clustering of occupied burrows, which is biologically more informative as these patterns represent the distribution of burrows selected by great gerbils from among those available to them. That there is clustering of occupied burrows indicates that the gerbils are indeed selective. Such clustering of occupied burrows was detected in 75% of the larger squares (> 590 m); empty burrows also showed evidence of clustering, although to a lesser extent. The basis for this selectivity is likely to be a combination of landscape‐ecological factors that influence habitat suitability, and aspects of gerbil dispersal behaviour linking currently occupied to newly colonized burrows. To explore these, it is necessary to understand the spatial scale at which clustering occurs.

Thus, in order to gain some sense of the validity of possible explanations, approximate estimates were made of the size of a typical cluster of occupied burrows. To do so, following Kiskowski *et al*. (2009), it is necessary to assume that clusters were circular and of equal diameter. Hence, estimates must be treated cautiously and only as supportive or not of particular possible explanations of the observed patterns. The average size of clusters can be estimated using the function $H=\sqrt{\left(K/\pi \right)}$ −s, where *K* is Ripley's *K* and *s* is distance (Kiskowski *et al*., 2009) and is equal to the distance *s* at which *H* attains its maximum. In our study, this is at *c*. 250 m (Appendix S1: Fig. S4). Again following Kiskowski *et al*. (2009), the inter‐cluster distance was found to be *c*. 550 m. It should be noted that clusters of occupied burrows are not ‘perfect’ clusters, in the sense that they do not consist of occupied burrows alone, which makes estimating exact cluster‐size difficult. It also should be noted that the size of clusters and therefore also inter‐cluster distance, will very possibly fluctuate with time, as the gerbil population size also fluctuates dramatically (Davis *et al*., 2004; Heier *et al*., 2011). One way to study this in the future is by using remote sensing. We have already shown that satellite images can be used to identify and localize burrows (Wilschut *et al*., 2013a). Currently, we are elaborating on this technique and preliminary results show that the occupancy status of a burrow can also be assessed based on satellite images, using normalized difference vegetation index (NDVI) values. This would allow the analysis of the distribution of occupied burrows in large areas and in multiple seasons.

At a larger scale, spatial autocorrelation in both burrow densities and occupied burrow densities was found for distances up to at least 7 and 2.5 km, respectively. This means that nearby areas have more similar (occupied) burrow densities than areas farther away. Previously, Wilschut *et al*. (2013a,b) studied burrow densities at large scales (three 20 km × 20 km areas) and also concluded that burrow densities were spatially structured. In the present study, the occupied burrow densities were found to be spatially structured as well. The auto‐correlation distance of occupied burrows will probably vary through the years and seasons, depending on whether the population is stable, increasing or decreasing. The distance at which there is autocorrelation of occupied burrows is relevant, because this determines how homogeneous or heterogeneous the abundance threshold for plague will be across the landscape.

Dealing first with habitat‐based explanation for the patterns, the landscape in the research area at small scales (i.e. the scale of squares: 200–1090 m) can vary quite substantially, especially in terms of vegetation density and topography. These small‐scale variations in habitat can lead to differing conditions for burrows. The clustering of occupied burrows is likely to be related to both selection of the most optimal burrows, and increased survival chances of gerbils in the more optimal burrows. However, the absence or presence of clustering was not found to be related to any of the environmental variables measured. One explanation for this might be simply a lack of statistical power (too few squares that were classified as clustered). Another possibility is the scale at which we tested the relationship between clustering and environmental variables. It might be that the factors we tested do influence occupancy, but play a role at the scale of the individual burrow, averaged out in our analysis at the whole‐square level. For example, work in progress focuses on habitat at the level of the individual burrow and finds that occupancy tended to be less likely on alluvial substrata, but that this was counteracted by the presence of dunes (B. Levick *et al*., in prep.).

The explanation for the larger scale spatial structures in both burrow and occupied burrow density is likely to be related to large‐scale variations in the landscape. In the case of burrow density, they may be related to soil texture, as this is thought to be an important aspect determining burrow density (Naumov & Lobachev, 1975). The occupied burrow density varied between 0 and 3 burrows ha^−1^ and also varied much in space and time. At larger scales, this variation can probably be explained by variations in vegetation density, which leads to differences in food availability.

Several aspects of great gerbil behaviour and ecology are also likely to contribute to the emergence of occupied burrow clusters. First, localized dispersal by great gerbil juveniles may reinforce local areas of high occupancy. Rates of dispersal by juvenile great gerbils are high, with one study reporting 42.8% of females and 100% of males changing burrows at least once within a year of their birth (Randall *et al*., 2005). The distances moved by juveniles are relatively short, however, averaging 125 m for females and 350 m for males; these distances conform well to the size of clusters found here. A high level of relatedness among adult females within burrows (Randall *et al*., 2005) also suggests that dispersal in this species reinforces pre‐existing social relationships. Second, predation may help limit the expansion of burrow clusters. Rogovin *et al*. (2004), for example, found that the frequency of visits by monitor lizards to a neighbouring colony depended on distance, with more distant colonies subject to a greater number of visits by lizards, and lower survival of juveniles and adult females, than colonies whose occupied neighbours were closer. Higher rates of predation on isolated burrows or those at the edge of clusters could arise from a reduced ability to benefit from the well‐developed alarm call system of great gerbils. Also, the risk of predation of an individual is enhanced when there are fewer nearby occupied burrows and gerbils. Next, predators may be less prone to errors in prey choice when the number of occupied colonies in an area is low. Predation may also serve to restrict dispersal distances, and thus the expansion of clusters, as there is a positive relationship between mobility and the probability of being killed (Norrdahl & Korpimäki, 1998).

The ability to detect clustering proved to be very dependent on the size of the research area. This is due to the fact that the clusters and the distances between them are fairly large, and it takes a large square to contain this pattern. Future research should focus on examining patterns in large research areas.

We have thus established that at a small scale, burrows occupied by great gerbils in the desert plague focus in south‐east Kazakhstan are clustered, and at a larger scale their densities are auto‐correlated. This population structure will probably influence the invasion and persistence of plague, suggesting in turn that exploring modifications to population structures in existing plague models is a priority. Many yet unexplained phenomena such as the long persistence of plague without being detected (Eisen *et al*., 2012) could perhaps be better modelled and understood using this knowledge.

## Biosketch

**Liesbeth Wilschut** is a landscape epidemiologist interested in how the landscape factors interact with hosts and vectors. She has been working primarily on plague in Central Asia for the past 3 years.

Author contributions: M.B., L.I.W. and A.L. initiated the research. L.I.W., A.L., N.K.H. and V.M.D. collected the data. L.I.W. carried out the analysis with help from S.E. and J.R. The writing of the article was led by L.I.W. and many contributions and edits were made by M.B., H.A.P., N.K.H., J.R., S.M.J., E.A.A., S.E., A.L. and V.M.D.

## Supporting information


**Appendix S1** Examples of distribution patterns.
