# Supplementary material for: Spatial distribution patterns of plague hosts: point pattern analysis of the burrows of great gerbils in Kazakhstan
Source: J Biogeogr. 2015 May 19;42(7):1281–92. doi: 10.1111/jbi.12534 (PMC4737218; doi:10.1111/jbi.12534)
Supplement: Supplementary file 1 — Appendix S1 Examples of distribution patterns. [file JBI-42-1281-s001.docx]

*Journal of Biogeography*

**SUPPORTING INFORMATION**

**Spatial distribution patterns of plague hosts: point pattern analysis of the burrows of great gerbils in Kazakhstan**

Liesbeth I. Wilschut, Anne Laudisoit, Nelika K. Hughes, Elisabeth A. Addink, Steven M. de Jong, Hans A.P. Heesterbeek, Jonas Reijniers, Sally Eagle, Vladimir M. Dubyanskiy and Mike Begon

**Appendix S1 Examples of distribution patterns**

**Figure S1.1** Two research squares with burrows of the great gerbils (*Rhombomys opimus*), and their corresponding *K*-values as a function of distance. *Left*: a typical research square that was classified as clustered. *Top left*: the occupied burrows (blue) and empty burrows (grey). *Bottom left*: the corresponding K-values (*K*_occ_), including the confidence interval (*K*_rs_) constructed by random sampling. *Right*: similar, but for a typical square classified as not clustered.

**Table S1.1** Results of the generalized linear models (GLMs), using all research squares. The five models with lowest AIC values are shown, ranked by AIC. Whether or not clustering of occupied burrows was present in a square depended mostly on the size of the research area and the number of occupied burrows and the number of burrows in the square.

| Variable | Intercept | Coeff. | P | Variable 2 | Coeff. | P | AIC |
| --- | --- | --- | --- | --- | --- | --- | --- |
| Size (m^2^) | -4.8 | 1.4e-05 | 0.002 | - | - | - | 40.7 |
| Size (m^2^) | -4.7 | -1.5e-02 | 0.007 | # occupied burrows | -1.62e-05 | 0.57 | 42.3 |
| Size (m^2^) | -5.04 | 1.2e-05 | 0.09 | # burrows | 9.64e-03 | 0.65 | 42.4 |
| # burrows | -4.54 | 0.034 | 0.001 | - | - | - | 43.1 |
| # occupied burrows | -3.6 | 0.05 | 0.0005 | - | - | - | 53.3 |
|  |  |  |  |  |  |  |  |

*
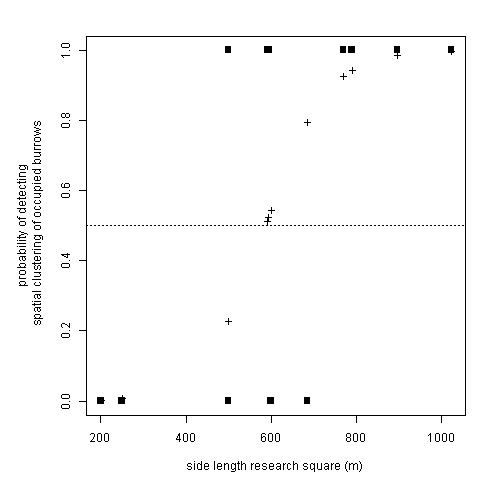
*

**Figure S1.2** S curve plot of the GLM, with the probability of detecting spatial clustering of occupied burrows on the y-axis and the side length of the research squares on the *x*-axis. The black squares represent the data points; the black “+” are the fitted points. The horizontal dotted line represents the 0.5 probability line of detecting spatial clustering of occupied burrows.

**Figure S1.3** *Top*: Histograms of burrow density data (left) and occupied burrow density data (right). *Below*: histogram of log occupied burrow density, which was used for the construction of the occupied density semi-variogram.

**Figure S1.4** Graph showing the values of *H* (equal to $\sqrt{(K/\pi)}\text{-}\text{s}$) for the squares with significant spatial clustering of occupied burrows. *H* can be used to deduce values for cluster size and inter-cluster-distance (Kiskowski *et al.*, 2009). Cluster size is approximately equal to the distance where *H* peaks, which is circa 250 m. The inter-cluster-distance is equal to twice the distance of the interval where *H* = 0. *H* is equal to zero around circa 50–100 m and around circa 375 m. The inter-cluster-distance will therefore be at least 275*2 = 550 m.

**References**

Kiskowski, M.A., Hancock, J.F. & Kenworthy, A.K. (2009) On the use of Ripley's K-function and its derivatives to analyze domain size. *Biophysical Journal,* **97,** 1095-1103.
